# Design of a Proteolytically Stable Sodium-Calcium Exchanger 1 Activator Peptide for *In Vivo* Studies

**DOI:** 10.3389/fphar.2021.638646

**Published:** 2021-06-07

**Authors:** Pimthanya Wanichawan, Jonas Skogestad, Marianne Lunde, Thea Parsberg Støle, Maria Stensland, Tuula A. Nyman, Ivar Sjaastad, Ole M. Sejersted, Jan Magnus Aronsen, Cathrine Rein Carlson

**Affiliations:** ^1^ Institute for Experimental Medical Research, Oslo University Hospital and University of Oslo, Oslo, Norway; ^2^ The KG Jebsen Cardiac Research Center and Center for Heart Failure Research, University of Oslo, Oslo, Norway; ^3^ Department of Immunology, Institute of Clinical Medicine, University of Oslo and Rikshospitalet Oslo, Oslo, Norway; ^4^ Department of Molecular Medicine, Institute of Basic Medical Sciences, Faculty of Medicine, University of Oslo, Oslo, Norway; ^5^ Department of Pharmacology, Oslo University Hospital Rikshospitalet, Oslo, Norway

**Keywords:** PLM, NCX1, disruptor peptide, NKA, cardiac, brain, peptide arrays, peptidomimetics

## Abstract

The cardiac sodium–calcium exchanger (NCX1) is important for normal Na^+^- and Ca^2+^-homeostasis and cardiomyocyte relaxation and contraction. It has been suggested that NCX1 activity is reduced by phosphorylated phospholemman (pSer68-PLM); however its direct interaction with PLM is debated. Disruption of the potentially inhibitory pSer68-PLM-NCX1 interaction might be a therapeutic strategy to increase NCX1 activity in cardiac disease. In the present study, we aimed to analyze the binding affinities and kinetics of the PLM-NCX1 and pSer68-PLM-NCX1 interactions by surface plasmon resonance (SPR) and to develop a proteolytically stable NCX1 activator peptide for future *in vivo* studies. The cytoplasmic parts of PLM (PLM_cyt_) and pSer68-PLM (pSer68-PLM_cyt_) were found to bind strongly to the intracellular loop of NCX1 (NCX1_cyt_) with similar *K*_*D*_ values of 4.1 ± 1.0 nM and 4.3 ± 1.9 nM, but the PLM_cyt_-NCX1_cyt_ interaction showed higher on/off rates. To develop a proteolytically stable NCX1 activator, we took advantage of a previously designed, high-affinity PLM binding peptide (OPT) that was derived from the PLM binding region in NCX1 and that reverses the inhibitory PLM (S68D)-NCX1 interaction in HEK293. We performed N- and C-terminal truncations of OPT and identified PYKEIEQLIELANYQV as the minimum sequence required for pSer68-PLM binding. To increase peptide stability in human serum, we replaced the proline with an N-methyl-proline (NOPT) after identification of N-terminus as substitution tolerant by two-dimensional peptide array analysis. Mass spectrometry analysis revealed that the half-life of NOPT was increased 17-fold from that of OPT. NOPT pulled down endogenous PLM from rat left ventricle lysate and exhibited direct pSer68-PLM binding in an ELISA-based assay and bound to pSer68-PLM_cyt_ with a *K*_*D*_ of 129 nM. Excess NOPT also reduced the PLM_cyt_-NCX1_cyt_ interaction in an ELISA-based competition assay, but in line with that NCX1 and PLM form oligomers, NOPT was not able to outcompete the physical interaction between endogenous full length proteins. Importantly, cell-permeable NOPT-TAT increased NCX1 activity in cardiomyocytes isolated from both SHAM-operated and aorta banded heart failure (HF) mice, indicating that NOPT disrupted the inhibitory pSer68-PLM-NCX1 interaction. In conclusion, we have developed a proteolytically stable NCX1-derived PLM binding peptide that upregulates NCX1 activity in SHAM and HF cardiomyocytes.

## Introduction

The cardiac sodium (Na^+^)/calcium (Ca^2+^) exchanger 1 (NCX1) plays an important role in the regulation of Na^+^ and Ca^2+^ homeostasis in the heart (Blaustein and Lederer, 1999; Shattock et al., 2015). Its activity is regulated by Na^+^ and Ca^2+^ concentrations and the membrane potential, in addition to several protein partners. During most of the excitation-contraction-relaxation cycle, NCX1 operates in forward mode, in which it transports one Ca^2+^ ion out of the cell in exchange for three Na^2+^ ions. However, during early systole, when the membrane potential exceeds the equilibrium potential of NCX1, the reverse mode is favored (Lines et al., 2006). This unique capability of NCX1 to work in both directions, confers its ability to regulate both relaxation and contraction during the excitation-contraction-relaxation cycle.

In cardiac disease, altered NCX1 activity and Na^+^ and Ca^2+^ homeostasis are often observed. The NCX1 Ca^2+^ removal rate may be slowed and in some pathologies it operates in reverse mode, leading to an increased cardiomyocyte Ca^2+^ influx and Ca^2+^ accumulation (Shattock et al., 2015). The intracellular Ca^2+^ accumulation is associated with an impaired diastolic function, accelerated myocardial necrosis, contractile failure and arrhythmias. Thus, because of its important role in the heart, NCX1 is considered to be a therapeutic target. However, it is an open question whether activation or inhibition of NCX1 would be beneficial to treat different types of heart failure (HF) (Shattock et al., 2015).

Our current understanding of NCX1 regulation in normal and pathophysiological conditions is limited. Several studies have indicated that NCX1 is modulated by β-adrenergic receptor activation, but there are conflicting reports as to whether or not protein kinase A (PKA) phosphorylates and exerts effects on NCX1 directly (Ruknudin et al., 2000; Wei et al., 2003; Ginsburg and Bers, 2005; Lin et al., 2006; Barman et al., 2011). We and others have shown that NCX1 can be phosphorylated by PKA and protein kinase C (PKC) at sites that are found in the cytoplasmic loop of NCX1 (Iwamoto et al., 1995; Iwamoto et al., 1996; Ruknudin et al., 2000; Wanichawan et al., 2011), but work in our laboratory has shown that PKA does not phosphorylate the full length cardiac NCX1 protein (Wanichawan et al., 2011).

Other components and proteins that are reported to regulate NCX1 activity are phosphatidylinositol 4,5-biphosphate (PIP2) (He et al., 2000), the endogenous exchanger inhibitory peptide (XIP) region (Matsuoka et al., 1997; Maack et al., 2005; Gök et al., 2020), calpain (Wanichawan et al., 2014), protein phosphatase 1 (PP1) (Hafver et al., 2016), and phospholemman (PLM) (Ahlers et al., 2005). PLM, which contains 3 PKA/PKC phosphorylation sites in its C-terminus (Walaas et al., 1994; Fuller et al., 2009), has been reported to be an endogenous inhibitor of NCX1 (Ahlers et al., 2005; Zhang et al., 2006; Wang et al., 2014). However, the concept of PLM regulation of NCX1 is a matter of debate. Although no transfer of fluorescence resonance energy (FRET) was detected between PLM and NCX1 that were tagged with fluorescent proteins (Bossuyt et al., 2006), we and others have shown that PLM co-precipitates with NCX1 in both cardiac (Ahlers et al., 2005; Wang et al., 2014; Wanichawan et al., 2016) and brain tissue (Wanichawan et al., 2016). It has further been shown that PLM binds directly to two regions of NCX1 that contain PASKT and QKHPD sequences (Zhang et al., 2011; Wanichawan et al., 2016) and that PLM is able to inhibit NCX1 activity when it is phosphorylated at serine 68 (pSer68) [or mutated to an aspartic (S68D) or glutamic acid (S68E)] (Zhang et al., 2006; Wang et al., 2014; Wanichawan et al., 2016). The level of pSer68-PLM appears to be upregulated in some animal HF models (Bossuyt et al., 2005; Hafver et al., 2016), but not in others (Shattock et al., 2015).

The effect of pSer68-PLM on possible NCX1 activity and Ca^2+^ handling is complex as pSer68-PLM concomitantly relieves the inhibitory effect on the Na^+^/K^+^-ATPase (NKA) (Cheung et al., 2013; Shattock et al., 2015; Yap et al., 2021), which leads to a decreased intracellular Na^+^ level, which in turn rather increases the driving force for Ca^2+^ efflux through NCX1. Whereas the cytoplasmic tail of the PLM oligomer is reported to bind to NCX1, the transmembrane domain of the PLM monomer binds to NKA (Shattock et al., 2015). Since PLM is an important regulator of NKA and possibly of NCX1, it is considered as a therapeutic target in cardiac disease, but few interventions are available.

In the present study, we aimed to analyze the binding affinities and kinetics of the possible PLM/pSer68-PLM-NCX1 interactions by surface plasmon resonance (SPR) and to develop a proteolytically stable, NCX1 activator peptide for future *in vivo* studies. For the latter, we took advantage of having access to a previously optimized PLM binding peptide (OPT, 20-mer). This peptide is derived from the QKHPD-containing region of NCX1 and has been shown to exhibit an 8-fold increase in pSer68-PLM binding compared with NCX1, and it reverses PLM (S68D) inhibition of exogenously expressed NCX1 in HEK293 cells (Wanichawan et al., 2016). The final developed NOPT peptide (16-mer), substituted with an N-methyl proline, showed a 17-fold increased stability in human serum compared with OPT, precipitated endogenous PLM from left ventricle lysate, exhibited a direct pSer68-PLM binding, outcompeted the direct PLM_cyt_-NCX1_cyt_ interaction and increased endogenous NCX1 activity when it was introduced as a cell-permeable peptide into adult cardiomyocytes that were isolated from SHAM-operated and aorta banded HF mice.

## Materials and Methods

### Peptide Array, Synthesis and Overlay

Peptides on cellulose membranes were synthesized using a Multipep automated peptide synthesizer (INTAVIS Bioanalytical Instruments AG, Koeln, Germany) (Frank, 1992, Frank, 2002). Peptides in solution were synthesized (listed below) at >80% purity by Genscript (Corporation, Piscataway, NJ, United States). For some of the peptides a cell-permeable TAT (RKKRRQRRR) or a biotin tag was included at either N- or C-terminus with or without an ahx-linker in between to avoid any potential steric hindrance.

PLM_cyt_: KRCRCKFNQQQRTGEPDEEEGTFRSSIRRLSSRRR

pSer68-PLM_cyt_: KRCRCKFNQQQRTGEPDEEEGTFRSSIRRLpSSRRR

OPT: YHPYKEIEQLIELANYQVLS

Mini-OPT: PYKEIEQLIELANYQV

NOPT: NMP-YKEIEQLIELANYQV

Scrambled control: VYEKNYLLLPAIEQEQSHIY (negative control peptide)

OPT (E6G): YHPYK **G** IEQLIELANYQVLS (negative control peptide)

OPT (1Y4A): YHPYKEIEQLIELAN **A** QVLS (negative control peptide)

Anti-NCX1 blocking peptide (amino acids 655–672): CGQPVFRKVHARDHPIPST

Peptide array membranes were blocked in 1% casein for 2–4 h and overlaid with 5 μM of biotinylated pSer68-PLM peptide in 1% casein overnight at 4°C. The membranes were then washed three times for 10 min in TBS-T before incubation with anti-biotin-horseradish peroxidase (HRP) for 1 h (see *Antibodies and Recombinant Protein*). Peptide-bound spots were detected as described in the immunoblotting section.

### Sample Preparation for Mass Spectrometry Analysis

Peptide stability was analyzed by incubating the peptide (300 μM) in 30% human serum (diluted in phosphate-buffered saline; PBS) (21792, Innovative research, MI, United States) at 37°C. Aliquots of 50 μl were collected at different time points (0–48 h) and thereafter trichloroacetic acid (TCA) was added to a final concentration of 5% to stop the reaction. The aliquots were kept at −20°C before centrifugation at 13,000 g for 15 min to remove the serum proteins. The supernatants were stored at −20°C until the mass spectrometry analysis.

### Mass Spectrometry Analysis

The peptides were purified using μC18 tips (Pierce C18 Tips, 100 μl) according to manufacturer’s instructions. 142 fmol purified peptide sample was analyzed by a nEASY-LC coupled to QExactive/QExactive Plus with EASY Spray PepMap®RSLC, C18, 2 μl, 100 Å, 75 μm × 25 column using a 60 min separation gradient (0–30% acetonitrile). MS raw files were submitted to MaxQuant software version 1.5.3.8 (Cox and Mann, 2008) for protein and peptide identification and intensity-based quantification. The search parameters were: First search error window of 20 ppm and main search error of 4.5 ppm; Unspecific enzyme; Minimal unique peptides 1; and FDR 0.01 (1%) for peptide and protein identification. Minimum and maximum peptide length for unspecific search were set to 8 and 25, respectively. The Uniprot human database was used (download from UniProt Sept. 2015) including the sequence for NOPT as the search database.

### Preparation of Rat Left Ventricle Lysates

Left ventricle lysates from 3–6 rats were prepared by using the TissueLyser II from Qiagen, Germantown, MD, United States. Briefly, frozen left ventricle tissue from rat heart (∼30 mg) was homogenized with ice-cold RH lysis buffer (PBS with 0.5% Triton and 0.1% Tween) supplemented with PhosSTOP tablets (4906837001, Roche Diagnostics, Indianapolis, IN, United States) and protease inhibitors (11836170001, Complete Mini EDTA-free tablets, Roche Diagnostics). The tissue samples were homogenized twice for 100 s at 30 Hz and then incubated on ice for 30 min. Supernatants were collected by centrifugation at 4,000 rpm for 10 min at 4°C and thereafter stored at -80°C. For IP experiments (Figures 3G, 4B), left ventricular tissue was pulverized in a mortar with liquid nitrogen, then added into lysis buffer (20 mM Hepes, pH 7.5, 150 mM NaCl, 1 mM EDTA**,** 0.5% Triton) with protease inhibitors and homogenized 3 × 1 min at 0°C. Supernatants were collected by centrifugation at 32,000 rpm for 60 min at 4°C and thereafter stored at -80°C.

### Pull-Down Assay With Biotinylated Peptides

Biotinylated peptide (8 μM final concentration) was incubated with 25 µl of monoclonal anti-biotin conjugated beads (A-1559, Sigma-Aldrich, St. Louis, MO, United States) in 100 µl of 1 × PBS for 2 h at 4°C with rotation. The beads were then washed one time with PBS, centrifuged at 1,000 rcf for 30 s at 4°C, and 200 µl of left ventricle lysate was added. The tubes were incubated for 2 h at 4°C with gentle rotation, and thereafter washed once in IP buffer (20 mM HEPES, pH 7.5, 150 mM NaCl, 1 mM EDTA and 1% Triton X-100). Finally, the samples were boiled in SDS loading buffer for 5 min at 96°C and analyzed by immunoblotting.

### Immunoprecipitation

Left ventricle lysate was incubated with the specific antibodies (2 μg/IP), 50 μl protein PLUS A/G agarose beads (sc-2003, Santa Cruz) and with or without presence of 100 μM NOPT-TAT or OPT overnight at 4°C. Immunocomplexes were washed twice in IP buffer (20 mM Hepes, pH 7.5, 150 mM NaCl, 1 mM EDTA**,** 1% Triton), centrifuged at 3,000 *g* for 1 min at 4°C and boiled in SDS loading buffer before the immunoblot analyses were undertaken. Equal amount of mouse IgG (sc-2025, Santa Cruz) or a custom made anti-NCX1 blocking peptide (CGQPVFRKVHARDHPIPST) incubated with anti-NCX1 prior to immunoprecipitation (Hafver et al., 2016) was used as negative control.

### Immunoblotting and Densitometry Analysis

The pull-down samples were separated on 4–15 or 15% SDS-PAGE Criterion Tris-HCl Gel (#3450027 and #3450020, BioRad Laboratories, Inc., Hercules, CA, United States) and transferred onto a PVDF membrane (1704157, Trans-Blot Turbo Mini PVDF Transfer Packs, Biorad Laboratories) using the Trans-Blot Turbo Transfer System (Biorad Laboratories). The PVDF was blocked in 1% casein or 5% milk in TBS-T for 60 min at room temperature (RT), and thereafter incubated with gentle agitation with primary antibody overnight at 4°C. After incubation with the primary antibody, the membrane was washed three times for 10 min in TBS-T and further incubated with the secondary antibody (see antibody section) and washed again. The chemiluminescence signals was developed using ECL Plus (RPN2232, GE HealthCare, United States) and detected by a LAS 4000 imager (Fujifilm, Tokyo, Japan). The densitometry analyses were performed using ImageJ (NIH).

### Antibodies and Recombinant Protein

Immunoblotting and IPs were carried out using anti-biotin-HRP (1: 5,000, A0185, Sigma-Aldrich, St. Louis, MO, United States), anti-PLM (anti-FXYD1, ab76597, 1:4,000, Cambridge, MA, United States), anti-NCX1 (1:1,000, custom made by Genscript Corp., Piscataway, NJ, United States, epitope: GQPVFRKVHARDHPIPST, mapped in (Wanichawan et al., 2011), anti-NKA (1:2,500, 05-369, Sigma-Aldrich) and anti-serine 68 phosphorylated PLM [pSer68-PLM, 1:1,000 (Fuller et al., 2009)]. Anti-rabbit IgG HRP (1:2,500 or 1:3,000, NA934, GE HealthCare, United States) and anti-mouse IgG HRP (1:3,000, NA931, GE HealthCare, United States) were used as secondary antibodies. Recombinant protein that covered the NCX1 cytoplasmic part added N-terminal histidine (HIS) and trigger factor (TF) tags to stabilize the protein (HIS-TF-NCX1_cyt_) was custom made by Genscript Corp., (Piscataway, NJ, United States).

### Enzyme-Linked Immunosorbent Assay

**FIGURE 1:**
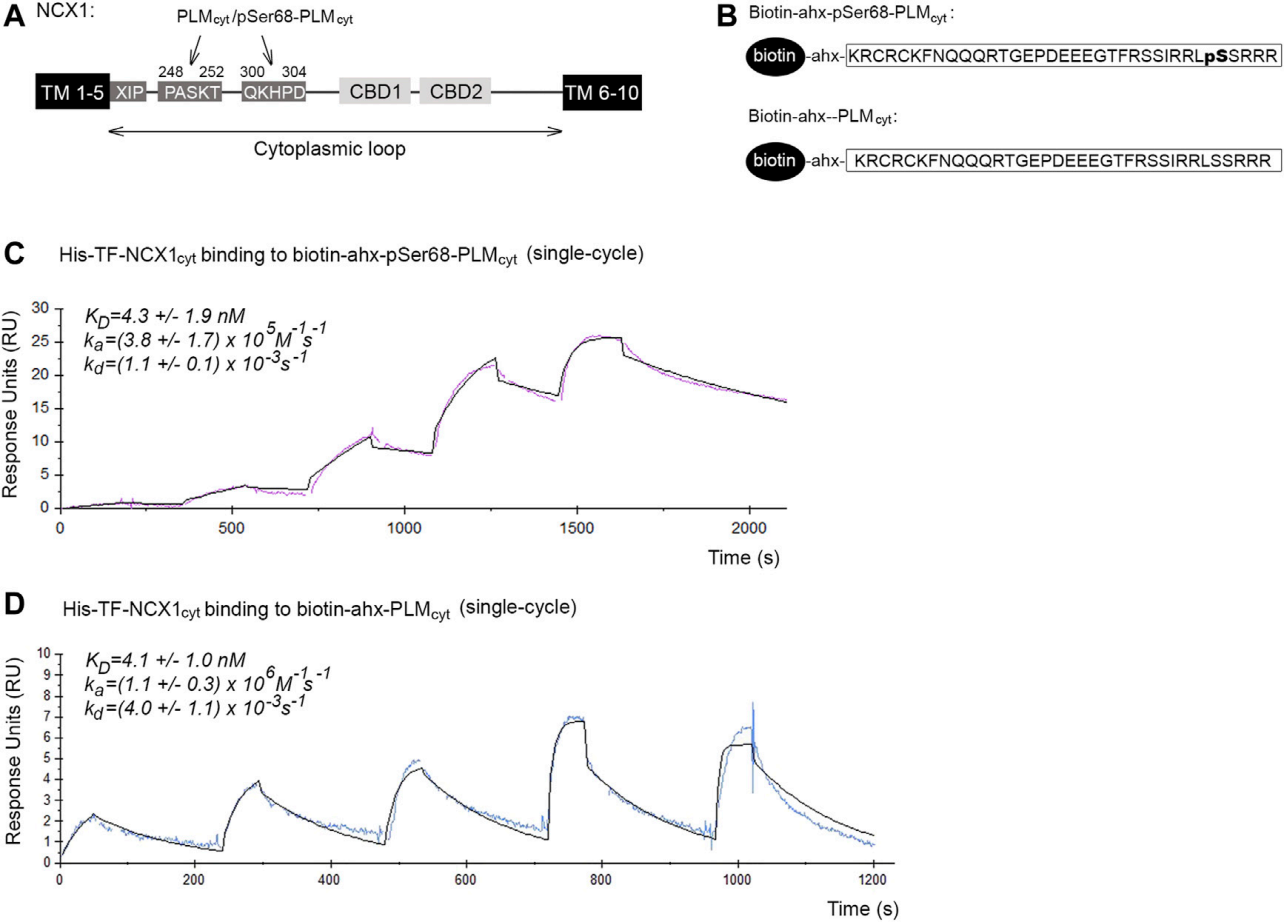
Binding affinities and kinetics of the pSer68-PLM-NCX1 and PLM-NCX1 interactions. **(A)** Illustration of the domain organization in NCX1. NCX1 contains 10 transmembrane (TM) domains, among which a large cytoplasmic loop between TM domains 5 and 6, that plays a regulatory role. The two PASKT- and QKHPD-containing regions in the cytoplasmic loop have been shown to bind directly to PLM/pSer68-PLM (Zhang et al., 2011; Wanichawan et al., 2016). XIP has been shown to bind to another site in the cytoplasmic loop, leading to NCX1 inactivation (Matsuoka et al., 1997; Maack et al., 2005; Gök et al., 2020). Binding of Ca^2+^ to CBD1 and CBD2 (calcium binding domain 1 and 2) leads to increased NCX1 activation. **(B)** Illustration of the two biotin-ahx-pSer68-PLM_cyt_ and biotin-ahx-PLM_cyt_ peptides that were used in Panel **(C,D)**, Figures 2B–E, 3F, and Supplementary Figure S1A. An ahx-linker was included to avoid any potential steric hindrance. SPR analyses of **(C)** biotin-ahx-pSer68-PLM_cyt_ and **(D)** biotin-ahx-PLM_cyt_ immobilized on SA chips and recombinant His-TF-NCX1_cyt_ protein injected at a range of concentrations over the chips. Red and blue show the experimental data in C (*n* = 3) and D (*n* = 5) (single cycles), respectively, whereas the black shows the fit (mathematical model).

A 96-well ELISA microplate was coated with 10 μg of PLM_cyt_ or pSer68-PLM_cyt_ (without biotin tag) (Figures 1C,D) in PBS and agitated gently overnight at 4°C. After this, each well was washed with PBS supplemented with 0.05% Tween (PBS-T), blocked with 0.5% gelatine (G-1890, Sigma) for 1 h at RT and incubated with 5 μl of the given peptides (final concentration: 50 μM) for 2 h at 37°C with gentle agitation. Each well was then washed five times with PBS-T, incubated with anti-biotin-HRP for 30 min at RT, and washed again five times with PBS-T, before incubation with 100 μl of the Ultra TMB substrate solution (34028, Thermo Fisher Scientific, Waltham, MA, United States) for 15–30 min at RT with gentle agitation (blue color development). In order to stop the reaction, 2N hydrochloric acid (HCl) was added into each well (yellow color). The absorbance of each sample was measured at 450 nm (Hidex Sense multimodal microplate reader, Åbo, Finland). For the competition experiment (Figure 1F), the microplate was coated with 10 μg/ml recombinant HIS-TF-NCX1_cyt_ protein before 0.5 μl biotin-ahx-PLM peptide (final concentration: 5 μM) was incubated with or without 10 μl NOPT (100 μM, without any biotin tag) for 2 h at 37°C with gentle agitation.

### Surface Plasmon Resonance Analyses

The SPR analyses were performed using Biacore X100 analyser (Biacore Inc., Uppsala, Sweden). The biotinylated pSer68-PLM_cyt_ or PLM_cyt_ peptides (ligand) were immobilized on streptavidin (SA) chips (BR100032, GE Healthcare Life Sciences) at ∼50–200 resonance units (RU) after the chip was conditioned through application of three consecutive 1 min injections of 1 x Biacore running buffer (BR100826, GE Healthcare Life Sciences). NOPT-TAT or recombinant His-TF-NCX1_cyt_ (analyte) dialyzed into the running buffer was diluted over a range of concentrations (NOPT-pSer68-PLM_cyt_: 4.5–175 nM; His-TF-NCX1_cyt_-pSer68-PLM_cyt_: 0.6–50 nM, 6.25–100 nM, and 3.12–50 nM; His-TF-NCX1_cyt_-PLM_cyt_: 9.38–150 nM, 12.5–200 nM, 18.75–300 nM, 4.69–75 nM and 9.38–150 nM), and injected over the sensor surface at a flow rate of 30 or 100 μl/min (to avoid mass transport limitations) for 180 s. The dissociation time was set at 600 s. The obtained sensorgrams were analyzed through use of Biacore X100 evaluation software and curve fitting was performed with the assumption of one-to-one binding (Langmuir) or steady state affinity.

### Heart Failure Model in Mice

8 weeks old C57BL6/J mice were randomized to SHAM or an aortic banding (AB) operation using o-rings (ORAB) as previously described (Melleby et al., 2018). Mice were anesthetized in a combination of 5% isoflurane and 95% O_2_, before endotracheal intubation and ventilation using a MiniVent rodent ventilator (Harvard Apparatus, United States). Left sided hemothoracotomy in the third intercostal space was performed, before dissection and placement of the o-ring around the ascending aorta. Animals were allowed to recover, and post-operative analgesia was achieved by injections of buprenorphine in accordance with the institutional guidelines. Animal experiments was approved by the Norwegian National Animal Research Committee (FDU 7040).

### Isolation of Adult Mice Cardiomyocytes

Cardiomyocytes were isolated based on a similar protocol as recently described (Ackers-Johnson et al., 2016). Mice were anesthetized and mask ventilated with a combination of 3% isoflurane and 97% O_2_. Deep surgical anesthesia was confirmed by abolished pain reflexes. The chest was opened, before the descending aorta and inferior caval vein were cut. Then 7 ml of buffer A (in mM: HEPES 25, NaCl 130, KCl 5.4, NaH_2_PO_4_ 0.4, MgCl_2_ 0.5, D-glucose 22, pH 7.4) with 5 mM EDTA was injected into the right ventricle. Thereafter, the aorta was clamped and the heart was excised. 10 ml of the buffer solution and thereafter 3 ml of the buffer solution without EDTA were injected into the left ventricle over 2–5 min. Then preheated solution A containing 0.8 mg/ml collagenase II was injected into the left ventricle over ∼20 min. The atria and right ventricle were removed, and the cardiomyocytes were isolated according to the same procedure as described for the Langendorff-based isolation of rat cardiomyocytes (described further below).

### Ca^2+^ Transients, SR Ca^2+^ Load and NCX Activity Measurements

Isolated cardiomyocytes were loaded for 10 min with 5 μM fluo-4 AM ester (Molecular Probes, Eugene, OR, United States) at RT. The superfusate contained (mM): NaCl 140, HEPES 5, KCl 5.4, CaCl_2_ 1, MgCl_2_ 0.5, D-glucose 5.5 and NaH_2_PO_4_ 0.4. The pH was adjusted to 7.4. The cells were loaded with either the TAT-conjugated NOPT (1 μM) or the TAT-conjugated scrambled control peptide (1 μM) for 20 min at RT. The peptides were also added to the superfusate. Whole-cell Ca^2+^ transients were recorded in field stimulated cardiomyocytes at 0.5 Hz (2.5 ms bipolar pulses) and 37°C with Cairn Research Optoscan Monochromator (excitation 485 nm, emission 515 nm long pass) (Cairn Research Ltd., United Kingdom). Regular Ca^2+^ transients were recorded for at least 3 min per cell, before the field stimulation was stopped and 10 mM caffeine was added. Background fluorescence was recorded for each cell. Ca^2+^ transients amplitude and SR Ca^2+^ load was calculated as maximal fluorescence (F) divided on resting fluorescence (F_0_) on field stimulated and caffeine evoked Ca^2+^ transients, respectively (See Figure 4I for illustration of protocol details). Tau values were obtained by mono-exponential fitting of the Ca^2+^ extrusion phase from regular transients (τ) and caffeine transients (τ_caff_) after rapid applications of 10 mM caffeine. The NCX rate constant was calculated as: $\;1/\tau_{\text{caff}}$ (Díaz et al., 1997), as the non SERCA2, non-NCX mediated Ca^2+^ removal was considered negligible.

### Isolation of Adult Rat Cardiomyocytes

Male Wistar rats were anesthetized in a chamber using inhalable isoflurane (Abbott Scandinavia Ab, Solna, Sweden), and 150 IE heparin was administrated intravenously for post-excision thrombosis prophylaxis. The heart was excised and immediately cooled in 0.9% NaCl at 4°C. The aorta was cannulated and the coronary arteries were retrogradely perfused in a modified Langendorff setup with buffer A (in mM: HEPES 25, NaCl 130, KCl 5.4, NaH_2_PO_4_ 0.4, MgCl_2_ 0.5, D-glucose 22, pH 7.4) at 37°C for 2–4 min, then with buffer A containing 0.8 g/L collagenase II (Worthington Biochemical Corporation, United States) and 6.7 μM CaCl_2_ for 18–22 min. Atria and the right ventricular free wall were removed before the left ventricle was cut into small pieces in 8–10 ml of the perfusate containing 500 μl 2% BSA, and mechanically isolated by careful pipetting with a Pasteur pipette for 1 min. The cardiomyocyte suspension was filtered through a nylon mesh (200 μm) (Burmeister), and left at RT for sedimentation. Immediately following sedimentation (∼5 min), the supernatant was removed. The cardiomyocytes were washed three times in buffer A containing 1) 0.1% BSA and 0.1 mM CaCl_2_, 2) 0.1% BSA and 0.2 mM CaCl_2_, and 3) 0.05% BSA and 0.5 mM CaCl_2_.

### Na^+^/K ^+^ -ATPase Activity Measurements

Ventricular rat myocytes were voltage clamped using continuous mode on an Axoclamp 2A amplifier and pCLAMP software (Axon Instruments, CA, United States). We used pipettes with moderately low resistance (2–3 MΩ) for a series resistance of 5–9 MΩ. The signal was sampled at 10 kHz and filtered with a low-pass filter before analysis. All amplifier and program settings were held constant during and between experiments. The cells were perfused at 37°C, and the perfusion system was placed very close to the cells, allowing rapid changes of solutions. We used an internal solution containing (in mM) (modified from (Despa and Bers, 2003): NaCl 20, KCl 30, K-Aspartate 80, TEA-Cl 20, HEPES 10, MgATP 5, MgCl_2_ 0.7 (free Mg^2+^ 1 mM using Maxchelator, Stanford), BAPTA 3, CaCl_2_ 1.15 (free Ca^2+^ 150 nM), pH = 7.2 (adjusted with KOH). 30 μM of OPT or the scrambled control peptide were added to the internal solution. After reaching whole-cell access, the cells were dialyzed for at least 4 min at −20 mV. Myocytes were patched in the following solution (in mM): NaCl 140, HEPES 5, KCl 5.0, CaCl_2_ 1.0, MgCl_2_ 0.5, D-glucose 5.5, NaH_2_PO_4_ 0.4, pH adjusted to 7.4 with NaOH. Solutions were switched after reaching whole-cell access to a solution containing (in mM): NaCl 20, NMDG 110, D-glucose 10, HEPES 5, TrisCl (or KCl) 15, NiCl_2_ 5, BaCl_2_ 2, MgCl_2_ 1, pH adjusted to 7.4 with HCl. Symmetrical Na^+^ solutions were used to minimize the effects of intracellular Na^+^ gradients. I_NKA_ was elicited by rapidly switching from a solution containing 0 mM K^+^ to a solution containing 15 mM K^+^; I_NKA_ was interpreted as the peak K^+^-sensitive current. Voltage ramp protocols were applied before and immediately after activation of I_NKA_. Cardiomyocytes were depolarized from −20 mV to + 70mV for 50 ms, then hyperpolarized to −120 mV (dV/dt = 380 mV s^−1^), and back to −20 mV. The difference between the ramp after activation of I_NKA_ and the baseline ramp defined the I_NKA_–voltage relationship. All currents were standardized by relating the absolute current (pA) to the cell size (pF).

### Ethics

All animal work was performed at the Institute for Experimental Medical Research, Oslo University Hospital, Norway. Animal handling was preapproved by the Norwegian National Animal Research Committee (FOTS ID 3820 for rats, and FOTS ID 7040 for mice) and conformed to the Guide for the Care and Use of Laboratory Animals (National Institutes of Health Publication 85-23, revised 2011).

## Results

### Surface Plasmon Resonance Analyses of the PLM-NCX1 and pSer68-PLM-NCX1 Interactions

To our knowledge, the binding affinities and kinetics of the possible PLM-NCX1 and pSer68-PLM-NCX1 interactions have not been investigated before. The cytoplasmic tails of PLM (PLM_cyt_) and pSer68-PLM (pSer68-PLM_cyt_) are reported to bind directly to two PASKT- and QKHPD-containing regions in the cytoplasmic loop of NCX1 (NCX1_cyt_) (illustrated in Figure 1A) (Zhang et al., 2011; Wanichawan et al., 2016).

To measure the binding affinities and kinetics of the pSer68-PLM-NCX1 interaction, a biotinylated peptide that covered pSer68-PLM_cyt_ (illustrated in Figure 1B, upper panel) was immobilized on a SA chip, and a range of concentrations of a recombinant polypeptide that covered NCX1_cyt_ was injected over the chip. The result was analyzed by fitting with a 1:1 interaction model (Langmuir). The K_D_ for the pSer68-PLM_cyt_-NCX1_cyt_ interaction was 4.3 ± 1.9 nM, with an association rate constant (k_a_) = (3.8 ± 1.7) × 10^5^ M^−1^s^−1^ and a dissociation rate constant (k_d_) = (1.1 ± 0.1) × 10^−3^ s^−1^ (Figure 1C). These results indicated a strong pSer68-PLM-NCX1 interaction.

NCX1 binding to PLM is thought to be independent of the phosphorylation status of the serine 68 (Wanichawan et al., 2016) Therefore, we also immobilized biotinylated PLM_cyt_ (illustrated in Figure 1B, lower panel) and injected a range of recombinant NCX1_cyt_ over the SA chip. Biotin-ahx-PLM_cyt_ was found to bind with similar affinity to NCX1_cyt_ (4.1 ± 1.0 nM) as the pSer68-PLM_cyt_ did, but showed higher association and dissociation rate constants (k_a_ = (1.1 ± 0.3) × 10^6^ M^−1^s^−1^ and k_d_ = (4.0 ± 1.1) × 10^−3^ s^−1^) (Figure 1D).

Taken together, our data indicated that both PLM_cyt_ and pSer68-PLM_cyt_ bound strongly to NCX1_cyt_, but the non-inhibitory PLM_cyt_-NCX1_cyt_ interaction exhibited higher association and dissociation rate constants (higher on/off rates).

### Identification of the Minimum pSer68-PLM Binding Sequence and of the N-Terminus as Substitution Tolerant

**FIGURE 2:**
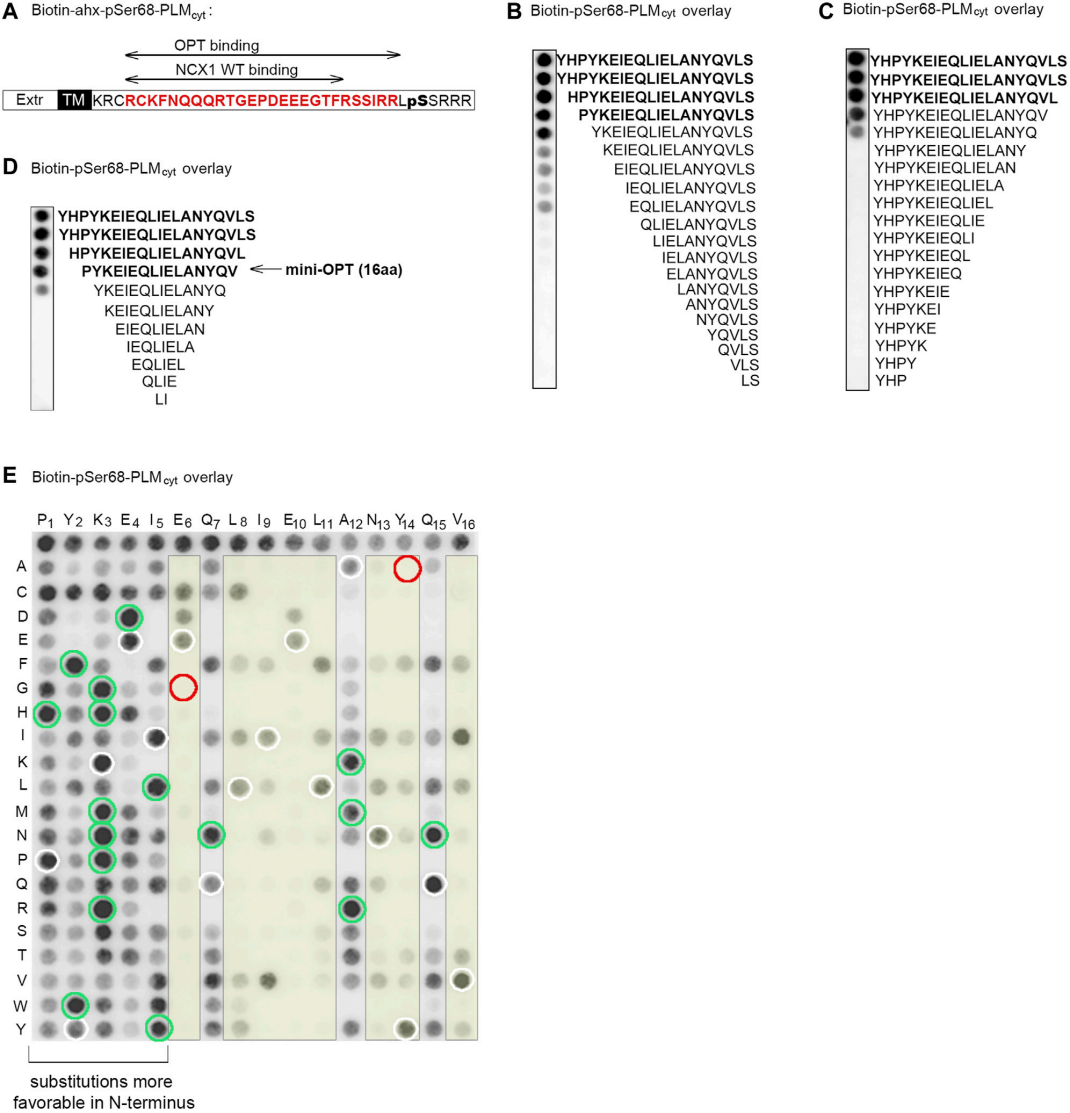
Identification of the minimal PLM binding sequence in OPT and of the N-terminus as substitution tolerant. **(A)** Illustration of the NCX1 and OPT binding sequences in PLM and pSer68-PLM cytoplasmic parts (the latter in red) (Wanichawan et al., 2016). **(B)** N-terminal, **(C)** C-terminal or **(D)** both N- and C- terminal truncations of the 20-mer OPT peptide (Wanichawan et al., 2016) were synthesized on membranes and overlaid with biotin-pSer68-PLM. Biotin-pSer68-PLM binding was detected with a monoclonal anti-biotin-HRP conjugated antibody. The minimal pSer68-PLM binding sequence was the 16-mer PYKEIEQLIELANYQV (mini-OPT) as indicated in **(D)**. **(E)** Amino acid substitutions of mini-OPT by two-dimensional peptide arrays aimed to identify modifiable positions. Biotin-pSer68-PLM binding was detected with a monoclonal anti-biotin-HRP conjugated antibody. Each residue in the mini-OPT sequence is written as a single-letter code above the array, whereas substitutions are given as single-letter codes to the left (vertically). The first row of the array shows biotin-pSer68-PLM binding to mini-OPT (*n* = 16). White circles show biotin-pSer68-PLM binding to mini-OPT dispersed through the array (internal control peptides, *n* = 16). Thus, in total, 32 spots showed biotin-pSer68-PLM binding to mini-OPT. Green circles indicate 17 substitutions that apparently enhanced peptide binding to biotin-pSer68-PLM. The E6G and Y14A substitutions (red circles) abolished pSer68-PLM binding and were used as negative control peptides in the pull down experiment in Figure 3C. The yellow boxes indicate positions that were less substitution tolerant than the others.

To develop a proteolytically stable NCX1 activator peptide, we used a previously designed high affinity PLM binding peptide (OPT: YHPYKEIEQLIELANYQVLS) that was derived from amino acids 301–320 in NCX1 that contained most of the QKHPD motif (Figure 1A) (Wanichawan et al., 2016). OPT has previously been shown to bind to the flexible region that is found upstream of serine 68 in PLM_cyt_ and to have a somewhat broader binding motif compared with that of the NCX1 region 301–320 (illustrated in Figure 2A, 41-RCKFNQQQRTGEPDEEEGTFRSSIRR-63, in red) (Wanichawan et al., 2016). OPT and NCX1 thus bind PLM independently of the phosphorylation status of serine 68.

To identify the minimal PLM binding sequence, we overlaid the biotinylated pSer68-PLM_cyt_ sequence (illustrated in Figure 1B, upper panel) onto N- or C-terminal truncations of OPT that was spot-synthesized on membranes. Immunoblotting with HRP-conjugated anti-biotin showed that OPT retained most of its pSer68-PLM_cyt_ binding capacity, although three or two amino acids were deleted at its N- (Figure 2B) or C-terminus (Figure 2C), respectively. We also performed N- and C-terminal truncations of OPT concomitantly (Figure 2D). Taken together, we identified the 16-amino acids sequence PYKEIEQLIELANYQV (mini-OPT) as the minimum sequence that was required to bind pSer68-PLM_cyt_.

To examine the substitution tolerance of this amino acid sequence, mini-OPT was analyzed by a two-dimensional peptide array, in which each amino acid (Figure 2E, sequence given above array) was systematically replaced with every possible other amino acid (Figure 2E, left of the array). When the membrane was overlaid with biotin-pSer68-PLM_cyt_ and HRP-conjugated anti-biotin was immunodetected, it was observed that positions 1–5 (P1, Y2, K3, E4, and I5), 7 (Q7), 12 (A12) and 15 (Q15) were tolerant of amino acid substitutions, whereas position 6 (E6), 8–11 (L8, I9, E10 and L11), 13–14 (N13 and Y14) and 16 (V16) were less tolerant (Figure 2E, yellow columns). Some of the substitutions in positions 1–5, 7, 12 and 15 appeared also to have a higher affinity for pSer68-PLM_cyt_ (Figure 2E, green circles) compared with the internal control peptides (Figure 2E, white circles), and were therefore tested in a second array. However, closer inspection revealed that these amino acid substitutions did not increase the pSer68-PLM binding further (Supplementary Figure S1A).

Conclusively, we identified PYKEIEQLIELANYQV (named mini-OPT) to hold the maximum pSer68-PLM_cyt_ binding capacity and to be substitution-tolerant at the N-terminus.

### Improvement of Peptide Stability and Retention of Capacity to Bind Phospholemman

**FIGURE 3:**
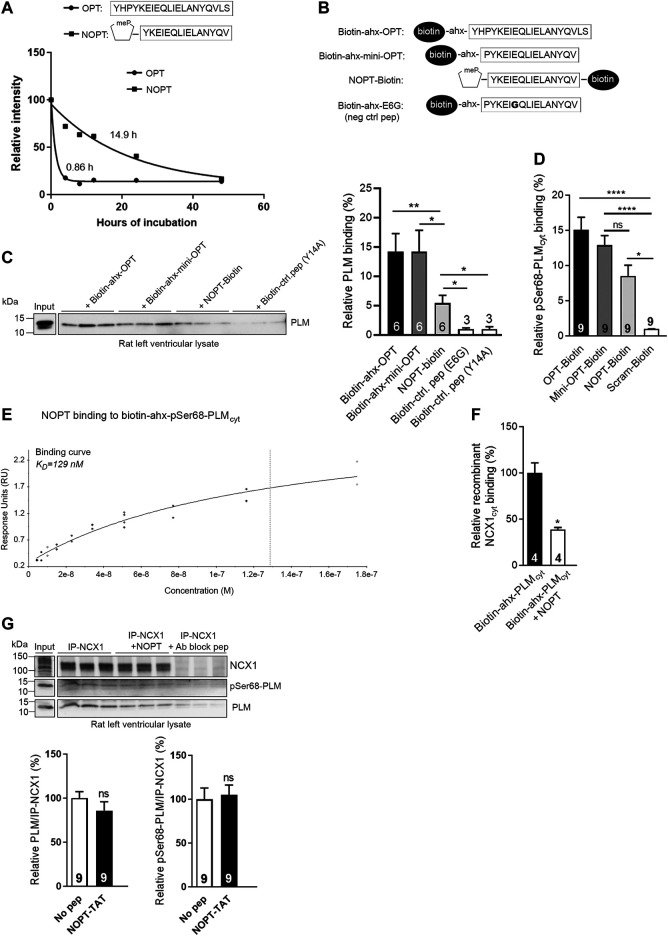
Analyses of peptide stability in human serum and PLM binding capacity of NOPT. **(A)** Stability of OPT (black circles) (*n* = 4) and NOPT (black squares) (*n* = 3–7) in 30% human serum analyzed by mass spectrometry. The relative intensity of the full length peptide/total intensity is given vs. hours of incubation and set to 100% at 0 h. **(B)** Illustration of the peptides used in the pull down experiment in **(C)**. **(C)** Pull down assay with biotin-ahx-OPT, biotin-ahx mini-OPT and NOPT-biotin in rat left ventricle lysate [peptides illustrated in **(B)**]. Except for NOPT, the biotin tag was added at the N-terminus of the peptides. An ahx-linker was included to avoid any potential steric hindrance. Endogenous PLM was detected with immunoblotting with PLM antibodies. *p < 0.05, **p < 0.01, examined by Mann-Whitney test (*n* = 3–6 rats, mean with SEM). Mini-OPT-E6G and mini-OPT-Y14A were used as negative control peptides (identified in Figure 2E, red circles). **(D)** Analyses of OPT-biotin, mini-OPT-biotin and NOPT-biotin binding to pSer68-PLM (coated in the wells and without a biotin tag) in an ELISA-based assay. Binding was detected with a monoclonal anti-biotin-HRP conjugated antibody following incubation with an Ultra TMB solution. *p < 0.05, ****p < 0.001, examined by one-way ANOVA with Tukey’s multiple comparison test (*n* = 9, mean with SEM). **(E)** Binding data of NOPT (4.5–175 nM) to biotin-ahx-pSer68-PLM_cyt_ immobilized on a SA chip (steady state affinity model). The data are obtained from two SPR analyses, and the blank subtracted sensorgrams are shown in Supplementary Figure S1B. **(F)** Biotinylated-ahx-PLM_cyt_ was incubated with or without excess NOPT (without any biotin tag) in wells coated with recombinant NCX1_cyt_ protein. *p < 0.05, examined by Mann-Whitney test (*n* = 4, mean with SEM). **(G)** Immunoprecipitation of endogenous NCX1-PLM/pSer68-PLM from rat left ventricle lysate with or without the presence of NOPT. The precipitates were analyzed by immunoblotting with specific antibodies against NCX1, PLM and pSer68-PLM. An NCX1 antibody blocking peptide (Ab block pep) was used as negative control. Ns, examined with unpaired *t*-test (*n* = 9, mean with SEM). Notably, densitometry analyses of the immunoblots indicated that five times more PLM co-precipitated with NCX1 (lane 1–3) compared with negative control (lane 7–9) (Supplementary Figure S1C).

N-methyl-amino acid substitutions have been shown to improve metabolic stability of peptides (Chatterjee et al., 2008; Ding et al., 2020). Therefore the N-terminal proline in the minimal pSer68-PLM_cyt_ binding sequence (mini-OPT) was substituted with an N-methyl-proline (NMP). This novel synthetic peptide was named NOPT. To analyze the stability of the peptide, we employed human serum which is the first compartment to which the peptides would be exposed during intravenous administration. Both the NOPT and OPT peptides (Wanichawan et al., 2016) (illustrated in the upper panel of Figure 3A) were incubated in 30% human serum and their degradation patterns were analyzed by mass spectrometry. Whereas the half-life of OPT was very short (0.86 h), the half-life of the smaller NOPT peptide was found to be 17 times higher than this (14.9 h) (Figure 3A, lower panel; MS data in Supplementary Tables S1–S6).

To confirm that the NOPT retained the PLM and pSer68-PLM binding capacities, several binding analyses were performed. First, a pull down experiment was performed with biotinylated peptides (which are illustrated in Figure 3B) in rat left ventricle lysate. Immunoblotting with PLM antibodies showed that NOPT-biotin precipitated endogenous PLM, although the binding capacity appeared somewhat reduced compared with that of biotin-ahx-OPT and the smaller biotin-ahx-mini-OPT (without the NMP substitution) (Figure 3C). Moreover, when peptides were used with a C-terminal biotin tag in an ELISA assay, it was confirmed that NOPT bound to pSer68-PLM nearly as well as the OPT and mini-OPT did (Figure 3D). Next, we analyzed the binding affinity of NOPT to pSer68-PLM_cyt_ by SPR. A range of concentrations of NOPT was injected over immobilized biotin-ahx-pSer68-PLM_cyt_ on a SA chip. Using the steady state affinity model, the dissociation equilibrium constant (K_D_) for the NOPT-pSer68-PLM_cyt_ interaction was found to be 129 nM (Figure 3E, the blank subtracted sensorgrams are shown in Supplementary Figure S1B). The dissociation rate (k_d_) and association rate constants (k_a_) were too fast to be determined. Despite its high on/off rates and lower binding affinity, excess NOPT reduced the biotin-ahx-PLM_cyt_-NCX1_cyt_ interaction with approximately 50% in an ELISA-based competition assay (Figure 3F). However, when applied to left ventricle lysate, NOPT was not able to outcompete the physical interaction between the two full length proteins (Figure 3G). This finding is in line with that NCX1 and PLM might be in larger protein complexes consisting of NCX1 dimers and higher order PLM oligomers (Beevers and Kukol, 2006; Bossuyt et al., 2006; Song et al., 2011).

Altogether, NOPT exhibited improved peptide stability, retained most of its PLM/pSer68-PLM binding capacities, and reduced the PLM_cyt_-NCX1_cyt_ interaction, but was not able to outcompete the physical interaction between the endogenous full length proteins.

### NOPT Increases NCX1 Activity in Adult Cardiomyocytes

**FIGURE 4:**
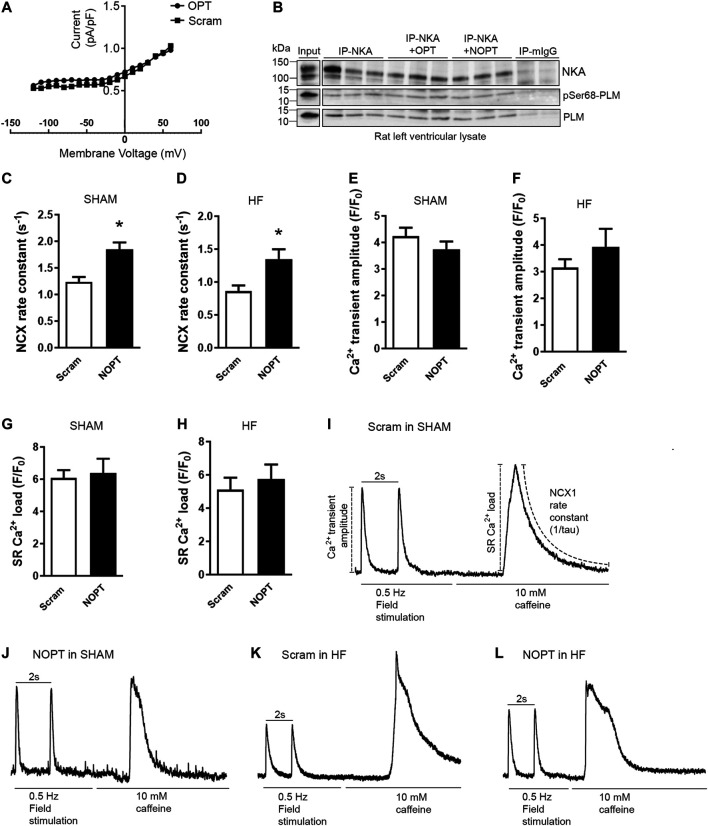
NOPT increases NCX-mediated Ca^2+^ extrusion in cardiomyocytes isolated from SHAM-operated and aorta banded HF mice. **(A)** No effect of the 20-mer original OPT peptide (Wanichawan et al., 2016) on NKA current was confirmed in adult rat cardiomyocytes. A scrambled control peptide was used as control (*n* = 6 cells from three rats). **(B)** Immunoprecipitation of NKA-PLM/pSer68-PLM from rat left ventricle lysate with or without in presence of OPT or NOPT (100 μM). The precipitates were analyzed by immunoblotting with specific antibodies against NKA, PLM and pSer68-PLM. Non relevant mouse IgG (mIgG) was used as negative control (*n* = 3). Average NCX rate constant measured in field stimulated **(C)** SHAM and **(D)** HF cardiomyocytes that were treated with NOPT-TAT or a scrambled control peptide. Average Ca^2+^ transient amplitude measured in field-stimulated **(E)** SHAM and **(F)** HF cardiomyocytes that were treated with NOPT-TAT or a scrambled control peptide. Average SR Ca^2+^ load from field stimulated **(G)** SHAM and **(H)** HF cardiomyocytes treated with NOPT-TAT or a scrambled control peptide. Totals of 10–13 cells from three mice were used in **(C, E, G)**, and 6–7 cells from three mice were used in **(D, F, H)**. *p < 0.05 vs scrambled control peptide examined with unpaired Student’s t-test (mean with SEM). Representative tracings of NCX1 activity in SHAM cardiomyocytes treated with **(I)** scrambled peptide or **(J)** NOPT-TAT. Representative tracings of NCX1 activity in HF cardiomyocytes treated with **(K)** scrambled peptide or **(L)** NOPT-TAT. All representative tracings **(I–L)** are normalized, and stimulation to evoke regular or caffeine induced Ca^2+^ transients is indicated for each cell. Illustration of analyses performed are shown in Panel **(I)**.

Initially and before we developed NOPT, we established that the OPT peptide did not have any effect on NKA activity in isolated adult rat cardiomyocytes (Figure 4A). Consistently, we found that presence of high concentrations (100 μM) of OPT or the smaller proteolytically stable NOPT did not affect the endogenous PLM/pSer68-PLM-NKA interaction in left ventricular lysate (Figure 4B). These findings are in line with that NKA binds to the transmembrane domain of the PLM monomer (Shattock et al., 2015), and not the cytoplasmic part (PLM_cyt_ and pSer68-PLM_cyt_) of the PLM dimer as NCX1.

We further analyzed the effect of NOPT on endogenous NCX1 activity in adult mouse cardiomyocytes. NOPT was made cell permeable by the addition of a TAT tag to its C-terminus. Compared with a TAT-tagged, scrambled control peptide, NOPT-TAT increased the activity of NCX1, as measured as the NCX1 rate constant, in adult cardiomyocytes that were isolated from both SHAM-operated (Figure 4C, approximately 40%) and aorta banded HF mice (Figure 4D, approximately 60%). NOPT-TAT did not change the Ca^2+^ transient amplitude or sarcoplasmic reticulum (SR) Ca^2+^ load in either SHAM (Figures 4E,G) or HF cardiomyocytes (Figures 4F,H). Representative tracings for NCX1 activity in SHAM and HF cardiomyocytes treated with the scrambled control peptide or NOPT-TAT are shown in Figures 4I–L.

Altogether, cell-permeable NOPT was found to increase endogenous NCX1 activity in adult cardiomyocytes that were isolated from both SHAM-operated and aorta banded HF mice. This finding suggests that NOPT is able to disrupt the inhibitory pSer68-PLM-NCX1 interaction.

## Discussion

In the present study, we aimed to analyze the binding affinities and kinetics of the possible PLM-NCX1 and pSer68-PLM-NCX1 interactions by SPR, and to develop a proteolytically stable NCX1 activator peptide for future *in vivo* studies. The cytoplasmic parts of PLM and pSer68-PLM interacted strongly with the cytoplasmic loop of NCX1, and showed similar *K*_*D*_ values of 4.1 ± 1.0 nM and 4.3 ± 1.9 nM, respectively. Although the binding affinities of the two interactions were similar, the non-inhibitory PLM_cyt_-NCX1_cyt_ interaction had higher association and dissociation rate constants (higher on/off rates) compared with those of pSer68-PLM_cyt_-NCX1_cyt_. These data strongly support previous findings that PLM and pSer68-PLM are direct NCX1 binding partners (Zhang et al., 2011; Wanichawan et al., 2016). However, an absence of interaction between PLM and NCX1 has also been reported (Bossuyt et al., 2006). In this latter study, the FRET reporter was cloned into the recently identified PLM binding region of NCX1 (Wanichawan et al., 2016), which might be a possible explanation for the non-detectable PLM-NCX1 interaction. False negative findings in immunoprecipitation experiments might also result if the PLM antibody epitope overlaps with the NCX1 binding site in PLM (41-RCKFNQQQRTGEPDEEEGTF-60) (Wanichawan et al., 2016). Such an antibody will only precipitate PLM without any NCX1 bound to it. Since the cytoplasmic part of PLM consists of only 36 amino acids, this likely applies to several PLM antibodies.

**FIGURE 5:**
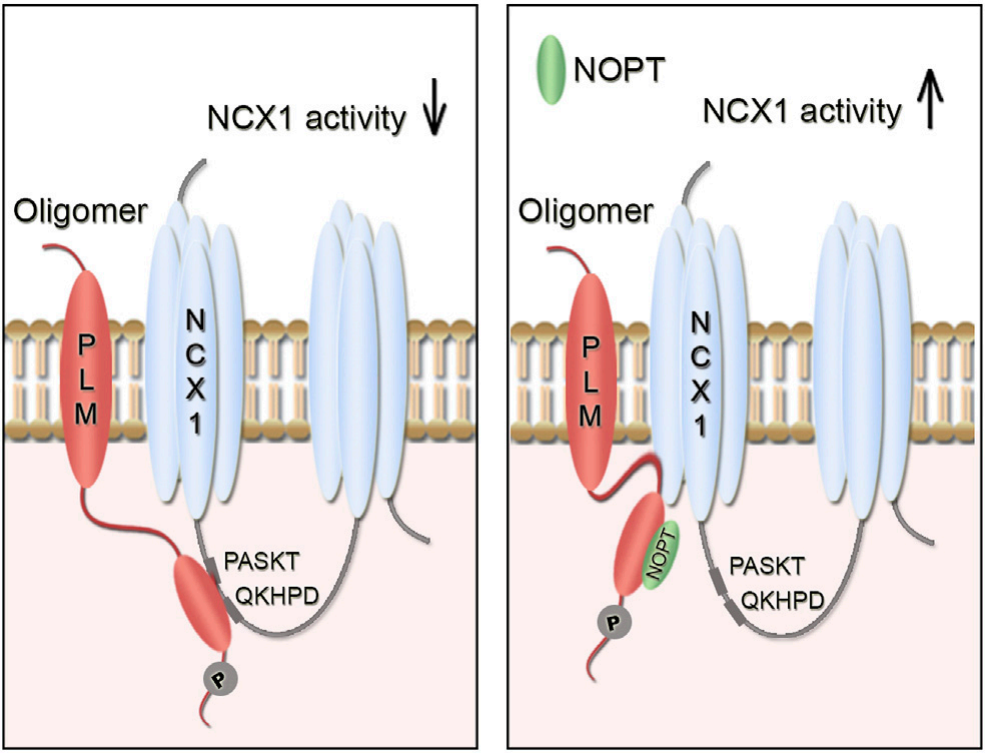
Illustration of the inhibitory pSer68-PLM-NCX1 interaction in heart and relief of its inhibition by NOPT. **(A)** Without NOPT, the pSer68-PLM dimer binds to the cytoplasmic part of NCX1 and inhibits NCX1 activity. **(B)** Binding of NOPT to PLM disrupts the inhibitory pSer68-PLM-NCX1 interaction, leading to increased NCX1 activation. NOPT does not disrupt the physical interaction between the endogenous full length proteins, which is in line with that NCX1 and PLM might be embedded in larger PLM-NCX1 protein complexes, consisting of NCX1 dimers (Ren et al., 2008; John et al., 2011) and PLM oligomers (Beevers and Kukol, 2006; Bossuyt et al., 2006; Song et al., 2011). It is also possible that two PLM molecules (dimer) bind to the two PASKT- and QKHPD-motifs in the NCX1 monomer.

To develop a proteolytically stable NCX1 activator peptide, we used a previously optimized PLM binding peptide (OPT, 20-mer), which had been shown to exhibit an 8-fold increase in pSer68-PLM binding affinity and to reverse PLM (S68D) inhibition of exogenously expressed NCX1 in HEK293 cells (Wanichawan et al., 2016). The final novel NOPT peptide (16-mer), which was the minimum pSer68-PLM binding sequence of OPT substituted with an N-methyl proline; exhibited a half-life of 14.9 h in 30% human serum, precipitated endogenous PLM from left ventricle lysate and was found to bind directly to pSer68-PLM_cyt_. Although NOPT exhibited a lower binding affinity than NCX1 to pSer68-PLM, excess NOPT was able to reduce the PLM_cyt_-NCX1_cyt_ interaction, suggesting that NOPT upon binding might induce a conformational change in PLM (or in its oligomerization state) that makes it less competent to bind NCX1. Compound induced conformational changes have also been reported for other proteins (Smith and Gestwicki, 2012). Notably, NOPT was not able to disrupt the physical interaction between the endogenous full length proteins, which is in line with previous studies reporting that NCX1 and PLM molecules probably are embedded in larger PLM-NCX1 protein complexes, consisting of NCX1 dimers (Ren et al., 2008; John et al., 2011) and higher order PLM oligomers (Beevers and Kukol, 2006; Bossuyt et al., 2006; Song et al., 2011). Identification of two PLM binding sites in the NCX1 monomer adds even more complexity to the PLM-NCX1 interaction (Zhang et al., 2011; Wanichawan et al., 2016). Importantly, cell-permeable NOPT-TAT increased the activity of NCX1 in adult cardiomyocytes that were isolated from both SHAM-operated and aorta banded HF mice, strongly suggesting that NOPT disrupts the functional inhibitory pSer68-PLM-NCX1 interaction (illustrated in Figure 5).

Since insulin’s first use as therapy in the 1920s, more than 150 peptides have been studied clinically and over 60 peptides have been approved for clinical use (Lau and Dunn, 2018). Most of these peptides have been developed for use within the fields of metabolic disease, oncology and cardiovascular disease, which are of great interest to the pharmaceutical industry. Several peptides are so-called replacement therapies that supplement peptide hormones in cases in which endogenous levels are inadequate, but peptides that disrupt protein-protein interactions have also entered the clinic (Stupp et al., 2014). Although most of the targets are extracellular proteins, such as G-protein-coupled and natriuretic peptide receptors, some are also directed against intracellular proteins and are delivered into the cell though application of cell-penetration strategies (Lau and Dunn, 2018).

Many cardiac proteins, such as NCX1 and PLM, play key roles in cardiovascular disease, making them attractive drug targets. Peptides that are derived from the binding surfaces of these proteins are potential drug candidates because they are easily synthesized and show high specificity and low toxicity. The binding affinities of such peptides to specific partners can also be optimized easily by amino acid substitutions (Carlson et al., 2006; Gold et al., 2006; Wanichawan et al., 2016). These peptides show close structural similarity to endogenous proteins and peptides, and therefore offer relatively low immunogenicity risks compared with other therapeutics (Ding et al., 2020). However, in general native peptides have short plasma half-lives due to the high concentrations of proteases in the human serum and rapid renal clearance and hepatic metabolism (Zaman et al., 2019). To overcome the problem with proteolytic degradation, incorporation of artificial amino acids, such as N-methyl amino acids, has been shown to improve peptide stability (Chatterjee et al., 2008; Ding et al., 2020). Consistently, it was found in this study that replacement of the N-terminal proline with an N-methyl-proline increased the stability of NOPT 17-fold *in vitro* (this study) compared with the OPT peptide (Wanichawan et al., 2016). This chemical modification affected only slightly the pSer68-PLM binding capacity of NOPT. Since the N-terminus of the peptide was identified to be the most substitution tolerant part of the molecule, we avoided any further chemical modifications either internally or at the C-terminus. Peptides can also be made more stable trough use of other strategies such as peptide cyclization (Ding et al., 2020) or to conjugate the peptide of interest to polyethylene glycol (PEG), lipids and proteins such as Fc fragments (Lau and Dunn, 2018). Modification of the peptide surface charge or increasing its hydrodynamic radius by conjugation to e.g. PEG or an unstructured polypeptide polymer, are strategies that can be used to reduce the renal clearance rate (Zaman et al., 2019). Ways to reduce renal clearance and hepatic metabolism of NOPT has to be determined in future studies.

The effect of NOPT *in vivo* remains an area for future investigation. We suggest that NOPT be delivered into animals continuously by use of osmotic mini-pumps. This strategy has been used recently to show the cardioprotection effect of a non-modified linear peptide with a half-life of 1.5 h (Altara et al., 2020). Increased NCX1 activity might not be beneficial in HF with reduced ejection fraction (HFrEF) where contractility is depressed. However, enhanced NCX1 activity and Ca^2+^ extrusion might be beneficial in subgroups of patients with HF with preserved ejection fraction (HFpEF), in which NCX1 activity has been reduced because of, e.g., increased and dominating Na^+^ levels (Eisner et al., 2020), and in HF that involves increased levels of pSer68-PLM (Bossuyt et al., 2005; Hafver et al., 2016). A study has supported the suggestion that increased NCX1 activity in HFpEF might have a beneficial effect. This study, of ventricular strips from failing human hearts, showed that the greater the expression of NCX1, the better was the diastolic function (Hasenfuss et al., 1999). Beneficial effects of increased NCX1 expression and activity have also been reported in several animal HF models. The induction of NCX1 overexpression in mice whose hearts had undergone pathological remodeling due to pressure overload was shown to reverse NCX1 activity to basal level and to prevent chamber dilatation with cardiac dysfunction (Ujihara et al., 2016). The cardiomyocytes that overexpressed NCX1 showed improved contractility, T-tubule integrity, and synchrony of the SR Ca^2+^ release and Ca^2+^ handling during EC coupling. Overexpression of NCX1 has also been shown to rescue postinfarction rat myocytes from contractile dysfunction (Zhang et al., 2002) and to attenuate myocardial dysfunction after infarction (Min et al., 2002). It is not clear whether the beneficial effects that were observed in these animal studies were due to the increased NCX1 protein levels, NCX1 activity or perhaps a combination of both; however, administration of NOPT into the animals could address this question in the future.

Finally, NOPT might also offer therapeutic potential in the brain, since PLM also interacts with NCX1 in this tissue (Wanichawan et al., 2016). The NCX protein family, which consists of the NCX1, NCX2 and NCX3 isoforms, has been shown to play a pivotal role in the maintenance of intracellular Na^+^ and Ca^2+^ homeostasis in the brain, and to have a protective role in neurodegenerative disorders, such as Alzheimer’s disease (Pannaccione et al., 2020) and brain ischemia (Molinaro et al., 2013). Moreover, overexpression of NCX1 and NCX3 has been shown to protect neurons in ischemic pre-conditioning (Pignataro et al., 2012).

Conclusively, in the present work described here, we identified strong pSer68-PLM/PLM-NCX1 interactions and designed a proteolytically stable NCX1 activator peptide (NOPT) for future *in vivo* studies. NOPT was found to precipitate endogenous PLM, bind directly to pSer68-PLM and reduce the PLM_cyt_-NCX1_cyt_ interaction and increase NCX1 activity in adult cardiomyocytes that were isolated from both SHAM-operated and aorta banded HF mice. NOPT might be a valuable tool to analyze the role of direct NCX1 activation *in vivo,* and to discover whether increased NCX1 activation is beneficial in different types of HF.

## Data Availability

All data required to evaluate the work are included in the article, and Supplementary Material. The mass spectrometry proteomics data have been deposited to the ProteomeXchange Consortium via the PRIDE (Perez-Riverol et al., 2019) partner repository with the dataset identifier PXD022797.

## Supplementary Material

Supplementary Figure 1(A) The 17 mini-OPT derivatives were analyzed for pSer68-PLM binding in a second peptide array and immunodetected with anti-biotin-HRP (lower panel). The relative pSer68-PLM binding was quantified by densitometry analysis and normalized to the average of OPT which was set to 1 (upper panel).**p* < 0.05, ***p* < 0.01, ****p* < 0.001, ^#^ *p* < 0.05 (mini-OPT versus I5L substitution), examined by ordinary one-way ANOVA with Dunnett’s multiple comparison test (*n* = 6, mean with SEM). **(B)** Blank subtracted sensorgrams of two SPR analyses of biotin-ahx-pSer68-PLM_cyt_ immobilized on a SA chip, and NOPT-TAT injected at a range of concentrations (4.5–175 nM) over the chip. **(C)** Densitometry analyses of the amount of PLM that co-precipitated with NCX1 compared with negative control (immunoblot is shown in Figure 3G **)**. ** p<0.01, examined by unpaired t-test (*n* = 3, mean with SEM).

## Abbreviations

HEK293, human embryonic kidney; HF, heart failure; HRP, horse radish peroxidase; Ka, association rate constant; Kd, dissociation rate constant; KD, dissociation equilibrium constant; NCX1, sodium-calcium exchanger 1; NCX2, sodium-calcium exchanger 2; NCX3, sodium-calcium exchanger 3; NKA, Na^+^/K ^+^ -ATPase; NMP, N-methyl-proline; NOPT, NMP-conjugated minimal pSer68-PLM binding OPT; OPT, optimized peptide (first generation); PIP2, phosphatidylinositol 4,5-bisphosphate; PLM, phospholemman; pSer68, phosphorylated serine 68; SA chip, streptavidin coated chip; SPR, surface plasmon resonance, TAT, transactivator of transcription (cell permeable tag); TM, transmembrane; WT, wild type; XIP, exchange inhibitory peptide.
