# Erratum: Design of a Proteolytically Stable Sodium-Calcium Exchanger 1 Activator Peptide for In Vivo Studies

**DOI:** 10.3389/fphar.2021.788046

**Published:** 2021-10-21

**Authors:** Frontiers Production Office

**Affiliations:** Frontiers Media SA, Lausanne, Switzerland

**Keywords:** PLM, NCX1, disruptor peptide, NKA, cardiac, brain, peptide arrays, peptidomimetics

Due to a production error, the incorrect **Supplementary Material** files were published. The Supplementary Material files have been corrected.

The publisher apologizes for this mistake. The original article has been updated.

