# Supplementary figures and images for: Design of a Proteolytically Stable Sodium-Calcium Exchanger 1 Activator Peptide for In Vivo Studies
Source: Front Pharmacol. 2021 Jun 7;12:638646. doi: 10.3389/fphar.2021.638646 (PMC8215385; doi:10.3389/fphar.2021.638646)

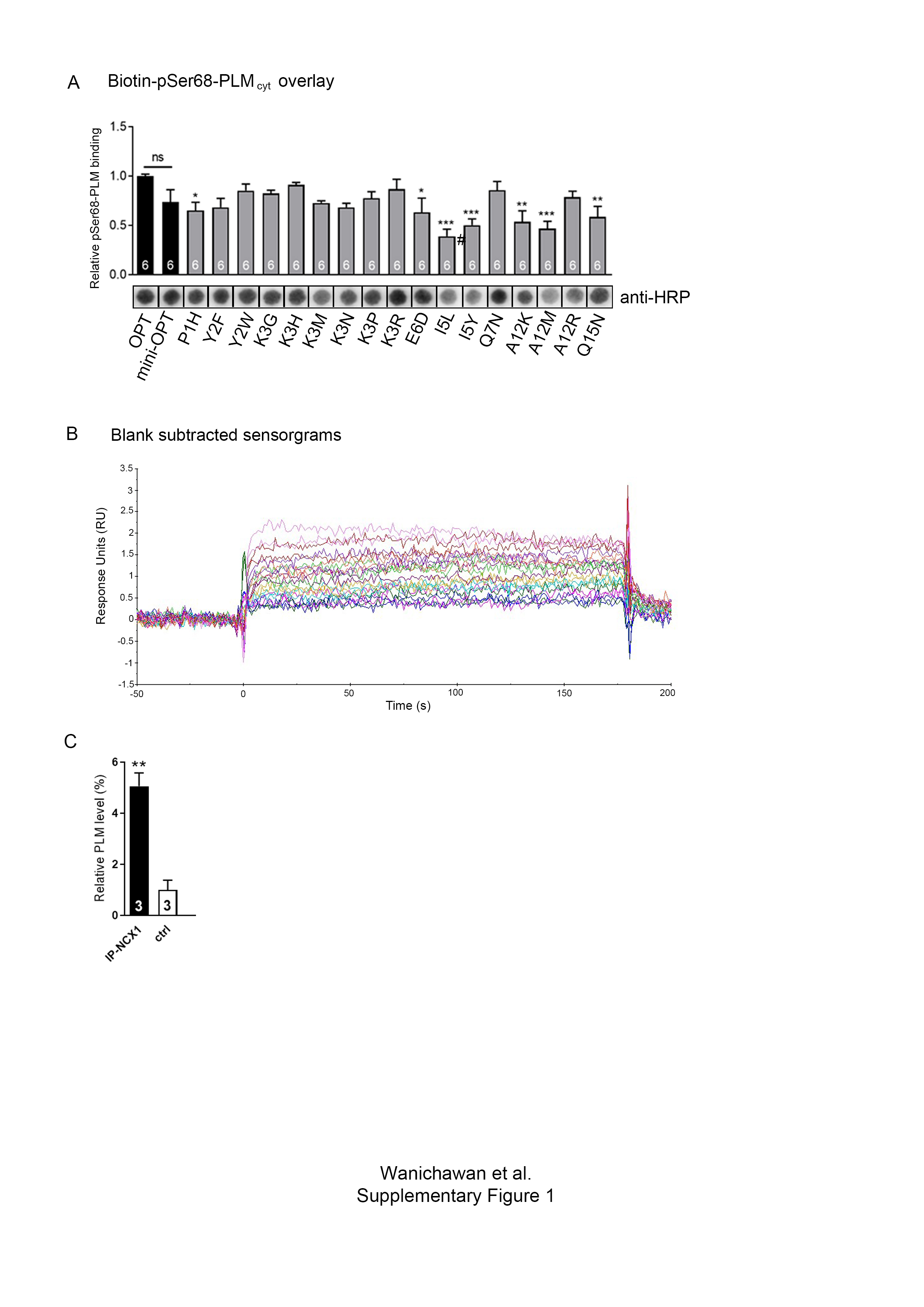

Supplement: Supplementary file 1 [file Image1.jpeg]
